# Evaluating the role that Care Groups play in providing breastfeeding and infant feeding support at community level: a qualitative study in Dedza district in Malawi

**DOI:** 10.12688/hrbopenres.13736.1

**Published:** 2023-09-04

**Authors:** Pieternella Pieterse, Aisling Walsh, Ellen Chirwa, Maria Chikalipo, Chimwemwe Msowoya, Janet Mambulasa, Anne Matthews

**Affiliations:** 1 Science & Health, Dublin City University, Dublin, Leinster, Ireland; 2 Royal College of Surgeons in Ireland, Dublin, Leinster, Ireland; 3 Department of Midwifery, Kamuzu University of Health Sciences, Blantyre, Malawi

**Keywords:** Care Groups, health policy, breastfeeding, community-based support, Malawi

## Abstract

**Background:** Promoting exclusive breastfeeding is a key nutrition policy objective in Malawi. This study assesses the role that care group volunteers (CGVs) play in providing breastfeeding and infant feeding support at community level. Care groups are a peer-to-peer learning approach, which has been part of Malawi’s nutrition policy since 2012, yet its efficacy and its role within community-level nutrition support remains under-researched.

**Methods:** In July 2021, we conducted 60 qualitative semi structured interviews in Dedza District with village leaders, Health Surveillance Assistants (HSAs), CGVs and district health officials, mothers with at least one child under two (n=36) who were randomly selected, and (purposively selected) mothers who had a child who was born prematurely or with low birthweight. The research was conducted in one of two care group programme implementation areas, or in several villages where care group interventions had not taken off. All interviews were recorded, transcribed and translated into English and then analysed using qualitative data analysis software. Thematic analysis was used to elicit key themes.

**Results:** Only eight out of 26 women in locations where care groups were active reported receiving breastfeeding support from care groups. All mothers reported receiving breastfeeding support at the health facility where they delivered their baby(ies) (n=36) and some (n=24) also at ante-natal care clinics. Where care groups were active, 18 out of 26 interviewees reported interacting with them, mostly during cooking demonstrations or receiving home visits. Little interaction was observed by interviewees between HSAs and CGVs (n=1) and no evidence suggested coordination between HSAs and CGVs around (vulnerable) newborn baby visits, as described by one HSA.

**Conclusions:** This research shows that care groups, despite being well-known, remain an under-appreciated and un-integrated volunteer cadre. Policy reform in relation to care groups in Malawi could improve care group efficacy.

## Introduction

In the past two decades, populations in many low- and middle-income countries (LMICs) have achieved significant reductions in child deaths ( Sharrow *et al.,* 2022; WHO, 2022). Health gains have been achieved due to greater investment in maternal and child health, leading to greater access to ante-natal care and increased facility-based deliveries. The increased uptake and availability of HIV and malaria testing and treatment methods have also significantly reduced child deaths ( Black *et al.,* 2019; Hemingway *et al.,* 2016; UNAIDS, 2022).

Malawi, a landlocked East-African country with a population of 19 million, has made significant gains in reducing deaths among children under five, achieving the child mortality-related Millennium Development Goal four ahead of schedule ( WHO, 2016). Between 1990 and 2020, infant mortality rates decreased from 143 to 29 per 1,000 live births, and neonatal mortality reduced from 50 to 19 per 1,000 live births ( Kanyuka *et al.,* 2016). Yet, for Malawi to achieve the Sustainable Development Goals (SDG) targets of neonatal and under five mortality rates, set at 12 and 25 per 1,000 live births respectively, further improvements are needed ( UN, 2022). The COVID-19 pandemic and climate shocks have recently added to Malawi’s challenges. Although COVID-19 is not thought to have affected under-five mortality significantly, increased levels of poverty due to ‘lock downs’, localised droughts and recent severe weather events are exacerbating Malawi’s already high levels of malnutrition, undernutrition, and stunting, which have all been identified as contributing to child mortality ( UNICEF, 2018; WHO, 2022). Encouraging the adoption of optimal infant nutrition practices, especially six months of exclusive breastfeeding (EBF) from birth, is therefore crucial. EBF for the first six months of a baby’s life greatly increases their wellbeing and can avert sickness and ill health. Worldwide, the scaling up of exclusive breastfeeding to near universal levels could prevent 823,000 annual deaths in young children ( Victora *et al.,* 2016).

EBF is strongly promoted in Malawi’s nutrition policies, which aim to prevent weaning before the age of six months, as recommended by the World Health Organisation ( WHO, 2021). In accordance with Malawi’s health and nutrition policies, pregnant women should attend at least four ante-natal care (ANC) visits at their nearest health facility, and the ANC curriculum should cover breastfeeding and the benefits of EBF ( GoM, DNHA, 2018). At the facility where women deliver, it is expected that women receive support with breastfeeding and are informed of the benefits of EBF, especially if it is their first baby ( GoM, MoH, 2009). Part of the duties of Malawi’s community-level health workers, the Heath Surveillance Assistants (HSAs) is visiting of all mothers within three days after they have arrived home with their newborn baby ( Guenther *et al.,* 2019), during which time it is expected that breastfeeding support will be provided and the benefits of EBF will be discussed. In addition, different volunteer cadres, including care group volunteers, are expected to actively support EBF at community level, as part of their promotion of key messages contained in Malawi’s health and nutrition policies.

The Care Group approach is a peer-to-peer learning model often used to promote good family health and child health practices at community level ( Perry *et al.,* 2015). In 2011, Malawi joined the Scaling Up Nutrition (SUN) movement and used its SUN membership to leverage support for the implementation of its National Nutrition Policy and Strategy Plan 2007–2015, which focused on broad-based nutrition interventions implemented at community level ( SUN, 2014). Malawi adopted the Care Group model ‘in order to roll out the Nutrition Education Communication Strategy (NECS) as an operational framework for the SUN strategy’ ( GoM, DNHA, 2017, p. 3). The Care Group model thus became a key community-level policy instrument in 2012, creating an “integrated, multi-sectoral approach to support communities and families” ( World Bank, 2019, p. 7).

The Care Group approach was originally designed in 1995 by staff of a non-governmental organisation (NGO) facing a significant lack of healthcare providers in the aftermath of the civil war in Mozambique. The Care Group’s peer-to-peer learning approach equipped mothers with basic hygiene and nutrition knowledge, and access to treatment for common illnesses, averting infant morbidity and mortality ( Davis *et al.,* 2013). The Care Group approach continues to be used by NGOs in locations where simple changes in behaviour (often improved day-to-day childcare and nutrition practices) can prevent adverse child health outcomes. The model works on the principle that in target localities, all women with children under two are enlisted into neighbourhood groups, and that per group of 10-12 women, one woman per group is elected Care Group volunteer. The care group volunteers (CGVs) receive monthly lessons (in groups of 10-12 CGVs) from a paid NGO staff member called a promoter. The CGVs pass these monthly lessons on to their 10-12 neighbourhood women’s group members. Well-implemented Care Group interventions ensure that all women with children under two in a certain target area receive the same guidance (on hygiene, nutrition, vaccinations, breastfeeding, weaning) at the same time, creating community-wide positive peer pressure to adopt new behaviours ( Laughlin, 2010).

The Care Group approach has been adopted in over 28 countries, and ‘Care Group-like’ adaptations have been implemented in additional LMICs, including interventions whereby the approach has been integrated into Ministry of Health systems ( Weiss *et al.,* 2015). The Care Group model, as adopted into national policy by Malawi’s Department of Nutrition, HIV and AIDS ( GoM, DNHA, 2012), was similarly modified to fit within the national structures. Malawi’s adoption of the care group model as a component of their national nutrition strategy, was inspired by the successful implementation of care group interventions by NGOs in Malawi in the early 2000s ( World Bank, 2019). In Malawi’s ‘modified Care Group’-policy, key responsibility for the training and supervision of the community-based volunteers, for example, rests with “frontline workers” according to the national Care Group guidelines ( GoM, DNHA, 2017, p. 5). While Malawi’s Care Group policy does not explicitly mention HSAs as the nominated frontline workers to support the Care Groups at community level, HSAs are the only cadre available at community-level, which makes their role and interaction with Care Groups important to examine ( Kok & Muula, 2013; Mziray *et al.,* 2017; Nsona *et al.,* 2012).

A reading of all health and nutrition policy papers since 2010, to seek references to the (adoption of the) Care Group approach in Malawi’s policies and strategies, reveals that the Malawi government’s guidance for the implementation of the Care Group approach has not been updated since 2012, when the Care Group approach was first adopted into policy. The integration and efficacy of Care Groups in Malawi has not been researched and published since the approach became part of Malawi’s nutrition strategy. This paper aims to address this knowledge gap.

### Aims and objectives

Our research project, focusing on Malawi’s health and nutrition policy implementation regarding exclusive breastfeeding and infant feeding support at community level, was guided by the following aims and objectives:

Aim: to examine the role that Care Groups play within Malawi’s breastfeeding and infant feeding support provision at community level.
Objective one: to explore how and from whom women received information about (exclusive) breastfeeding - before delivery, immediately after delivery and in the days and weeks thereafter.
Objective two: to evaluate, in places where Care Groups operate, if and how CGVs played a role in visiting new mothers to support infant feeding
Objective three: to explore if CGVs appeared to be working in an integrated manner with HSAs or other community-level structures, as Malawi’s national policy dictates.

## Methods

In advance of the field research, we conducted a realist synthesis of care group literature ( Pieterse *et al.,* 2021), which informed the interview guides that were used during the empirical research described in this paper.

### Ethical statement

Ethical approval for this research was obtained from Dublin City University Research Ethics Committee on the 11th January 2021 (approval reference: DCUREC/2020/268) and from the College of Medicine Research Ethics Committee (COMREC) on the 4th of February 2021 (approval reference: P.02/21/3266). During the application for ethical approval, the researchers were directed by COMREC to pay each interviewee the equivalent of US$10 in Malawi Kwacha in cash ( Nyangulu *et al.,* 2019), with which we complied. Written informed consent was obtained from all interviewees.

### Study design

For the field research, we used a qualitative approach, conducting one-to-one interviews in Dedza District. The research focused firstly on mothers with children under two, who were likely to have recently received breastfeeding and infant feeding support. To examine the role that healthcare providers, community leaders and district health officials’ play in supporting and integrating Care Groups into the communities, we interviewed village headmen, HSAs and a number of district- level health officials, as well as an NGO representative. Using focus group discussions was ruled out to reduce the risk of COVID-19 and associated restrictions in place at the time of data collection.

The Care Group interventions that were the focus of this study were implemented by an international NGO that had been actively implementing the SUN programme in Dedza since 2012. Their willingness to facilitate the research and the opportunity this offered to study two Care Group interventions led us to focus on these particular interventions. Both interventions used the Care Group model. One intervention was implemented across the entirety of Dedza District, while the second intervention, providing additional support to Care Groups volunteers, was limited to half of the district’s traditional authorities (TAs). In advance of the field research, the implementing NGO explained that since they supported the establishment of Care Groups in Dedza in 2016, several communities had witnessed a collapse of care group activities, due to the fact that despite repeated efforts to train and incentivise, volunteers were unwilling to carry out the tasks expected of them. This provided us with the opportunity to explore the support for EBF in areas where no Care Groups were operational.

We made efforts to identify Care Group interventions that were established without the support of an international NGO or donor-funded intervention but were unable to find any that we could include in our research. A report commissioned by the Department of Nutrition, HIV and AIDS at the time of our research was to demonstrate that very few Care Groups have been started without external support in Malawi ( GoM, DNHA, 2021), despite the national guidelines, which set out how this can be done.

### Research setting

Our field research was conducted in Dedza District in the Central Region of Malawi. Poverty remains widespread in Malawi, where 61.7% of the population was reported to be multi-dimensionally poor, according to a 2021 research report ( NSO *et al.,* 2021). 20% of the country’s population live in extreme poverty, defined as an inability to satisfy basic food needs, while 70.3% live below the international poverty line of $1.90 a day ( World Bank, 2020). Food insecurity is high, and a significant portion of Malawi’s population require food assistance annually, with malnutrition remaining a challenge nationally as a major contributor to preventable child deaths in Malawi ( UNICEF, 2019). Dedza District has higher than the average mortality rates for the Central Region with a neo-natal mortality rate of 33 deaths per 1,000 live births and an infant mortality rate of 53 deaths per 1,000 live births, compared to national rates of 19 and 29. In Dedza, 18% of children are born with a low birth weight (below 2.5kg), compared with national average of 12.3% ( NSO & ICF, 2017).

### Sampling and data collection

In order to answer our first and second research objective, ‘to explore how and from whom women received information about (exclusive) breastfeeding - before delivery, immediately after delivery and in the days and weeks thereafter’ and ‘to evaluate, in places where care groups operate, if and how CGVs played a role in visiting new mothers to support infant feeding’, we interviewed mothers with children under the age of two (including a total of nine women who had a child who was born prematurely or with low birthweight) and asked her primarily about the support for EBF that she may have received after the delivery of her most recent baby/ies. We also interviewed other community members, including CGVs, HSAs, and District Health Authority personnel, in order to explore (research objective three) ‘if CGVs appeared to be working in an integrated manner with HSAs or other community-level structures, as Malawi’s national policy dictates’. The interviewees at district-level were identified by the District Medical Officer, to whom the research team first reported to introduce themselves at the start of the data gathering visit in Dedza District. Standard operational procedures were developed for the researchers to guide purposive sampling. The research team were to arrive at a study site, find and interview the Village Headman, ask to be introduced to a HSA, interview the HSA, and then ask the HSA to bring them to houses of women who had a child under two years of age. The number of women who fitted the research participant criteria and were willing to be interviewed at each location, determined the total number of interviews conducted.

In total 36 women with at least one child under two were interviewed.

An overview of research participants: 

**Table:** 

	Care Group intervention A	Care Group intervention B	Location C- No Care Groups
Mother with child(ren) under two	8	10	10
Mother of ‘low birthweight’ baby or baby born prematurely (before 37 weeks of gestation)	3	5	0
Village leader	2	2	2
HSA	2	1	2
Care Group staff/ volunteer	5	4	0
District Staff	3 District Headquarter Town		
NGO staff	1 District Headquarter Town		

Each of the women was interviewed using semi-structured interview guides, which were prepared in English and then translated into Chichewa by the researchers who conducted the interviews. The guides were developed from our aims and objectives and drew on a realist synthesis of Care Group literature, they ( Pieterse *et al.,* 2021) ^27^ were piloted with five women before the field research took place. The original interview guides were shortened, and some language was simplified after two members of the research team (MC and JM) used them to interview five women with children under two, in two villages close to Blantyre.

All data were collected by three members of our research team (EC, MC and CM), who were joined by two additional research assistants (JM and JC). All researchers were Malawian and used Chichewa to conduct the interviews, using the Chichewa version of the interview guides. Interviews were audio recorded and later transcribed and translated into English. The audio recordings were deleted off all recorders after transcription. The translations were conducted by all Malawian research team members, who double checked each other’s translations against the Chichewa transcriptions.

### Data analysis

Two researchers (PP and JM) coded all data using qualitative data analysis software Nvivo version 12 ( https://lumivero.com/products/nvivo/). (A freely accessible software package, Taguette ( https://www.taguette.org/), could also be used to run the same analysis), ensuring that 15% of the interviews were coded by a second person (any member of the research team) during the first round of inductive, open coding. We used Braun and Clarke’s ( 2006, 2021) approach to thematic analysis to guide our multiple rounds of coding: The manuscripts were read several times to familiarise ourselves with the content. The first round of coding was done inductively, where all interesting and relevant sections of text were coded openly, without any guidance, codebook or preconceived structure. The generated codes were them loosely grouped. The second round of coding focused on the development of themes, based on the open codes generated in the first round of coding. Team discussions took place to agree on the formation of themes. Further coding took place to explore differences in the characteristics or themes across the different interventions or across variations in context ( Bazeley, 2009). The codes were then agreed upon by the research team and named and ordered into several clusters of related findings. This allowed for the writing of a feedback report for the NGO that facilitated the research (available on the project website), and academic publications. The codebooks are available in the open access data repository established for this research. Table 1 shows the trustworthiness table for the qualitative data.

**Table 1.** Trustworthiness in qualitative data.

Criterion	Strategy	Actions
Credibility	Prolonged engagement (via Care Group implementing NGO)	NGO has worked in this area since 2012
	Peer briefing	Daily debriefs during data gathering phase, full research team debriefing after field research
	Triangulation	Cross checked with previous NGO reports of this intervention, NGO staff interviews, other Care Group literature
Transferability	Providing thick description	Conducted pre-study assessment, key-informant interviews
	Purposive sampling	Worked with local Health Surveillance Assistant to ensure sampling was done correctly
Dependability	Creating an audit trial for data	Used primary and secondary data to contextualise outcomes
	Triangulation	Cross checked with previous NGO reports of this intervention, NGO staff interviews, other Care Group literature
Confirmability	Reflexivity	Used primary and secondary data to anticipate outcomes, contextualise findings
	Triangulation	Cross checked with previous NGO reports of this intervention, NGO staff interviews, other Care Group literature

*Based on: Huberman AM, Miles MB. Data management and analysis methods. In: Denzin NK, Lincoln YS (editors). Collecting and interpreting qualitative materials. Thousand Oaks, CA: Sage Publications, Inc, 2000; p.179-210.*

## Results

### Sources of breastfeeding support (objective one)

Overall, in the areas where Care Groups were purportedly active, only eight out of 26 interviewees (women with children under two) reported receiving breastfeeding support from Care Groups. Those who received Care Group support reported anything from having been encouraged to breastfeed exclusively during a group meeting, to home visits whereby CGVs engaged with a mother and her newborn baby on a one-to-one basis:

“Q: What materials did care groups use when they come to visit at home?
A: They had books; the books had pictures which they could show us and there were some explanations below to tell us what we can do with our babies when breastfeeding.
Q: How many times has the care group visited you since the birth of your baby?
A: Since my baby was born, they visited me twice. They first visited me when the baby had just born to advise me on how I can exclusively breastfeed my baby until she reached 6 months.” [03MCA].

**Figure 1.**
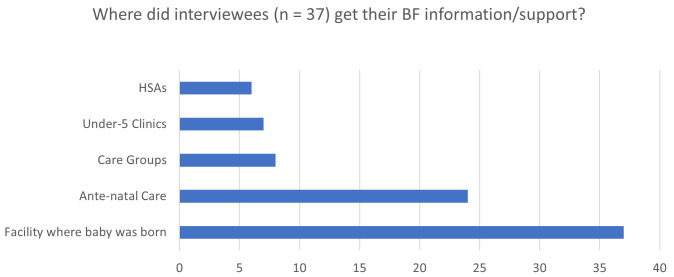
Source of breastfeeding support (outside of family and friends).

All 36 women who were interviewed reported delivering their baby at a health facility. On the question regarding where, or from whom, women received breastfeeding support (excluding support from family and friends), all women reported receiving some support at the facility where the baby was born, ( Figure 1). ‘Support’ was often no more than an instruction from somebody at the health facility that a mother should breastfeed her newborn infant; this was interpreted as support: ”At that time, they the nurses said the baby was hungry and needed to feed; she also said that the baby was cold so I needed to warm her.” [04MCA].

24 out of 36 women also reported receiving EBF support during their ante-natal visits:

“A: We were listening to the advice when we come to the clinic the ante-natal clinic.
Q: What did they say?
A: That we should exclusively breastfeed; if the baby is sleeping, when she wakes up we should breastfeed her and after some time we should breastfeed the baby again so that the baby should grow healthy she should not become malnourished.” [35PLBWB].

Seven women mentioned receiving EBF support during the ‘under-five clinics’ (monthly baby check-ups usually conducted by a health facility out-patient team that provides vaccinations, growth monitoring, etc.) and six out of 36 women reported receiving support from a HSA. From the responses it appears that the HSAs tended to be the individuals who conducted the under-five check-ups at the local clinics, so it is hard to know whether ‘receiving support from the HSA’ means during the under-five check up at the local clinic, or during a home visit. In some locations both seem to happen, in other places, interviewees only reported seeing HSAs when they brought their babies for vaccinations and check-ups:

“Q: What about HSAs; do they come here to counsel women?
A: At the under-five clinic; if there is an under-five clinic then they provide the counselling.
Q: What about when there is no clinic?
A: No they don’t.
Q: So what do they talk about at the clinic?
A: They talk about the breastfeeding.” [29PLBWA].

Non-professional support for breastfeeding was provided most often by mothers or mothers-in-law (n=19), followed by fathers (n=17), who were often mentioned as being supportive, taking on chores, and reminding women to make sure the baby gets breastfed often. Other family members (n=14) were also mentioned as providers of breastfeeding support, while neighbours and friends did less so (n=8). All women mentioned at least one source of ‘non-professional’ EBF support, some mentioned two or three.

### Care Group activities (objective two)

The Care Groups in Dedza work with NGO promoters, who train CGVs. CGVs pass on the promotors’ health and nutrition lessons on to so-called ‘cluster leaders’, who are tasked with home visits. Findings show that 18 out of the 26 interviewees living in areas with Care Groups mentioned interacting with them. Home visits were mentioned, as were group activities. The group activities, especially cooking demonstrations, appear to have been well received by the women who were interviewed. Several women credited Care Group cooking demonstrations for teaching them how to add nourishing ingredients to the porridge they feed their small children. Some linked this to their infants’ improved growth [18MCB] and mentioned learning “from the group”. Group meetings that involved cooking and sharing food appeared to live up to some mothers’ expectations of what NGO-supported interventions do, while receiving counselling, or information, only, was not always appreciated in the same manner. A CGV recounted being told by a family “we don’t see a reason why you come to visit us because there is nothing that you give us” [52CGVA], while several mothers explained during our interviews that they had received ‘no support’ from Care Groups, which later turned out to mean no material support, while the same women further expressed their satisfaction about the advice they have received from Care Group cluster leaders or volunteers [e.g. 02MCA]. 

### Care Group interaction with HSAs and District officials (objective three)

The CGVs, cluster leaders, and promoters interviewed for this research did not make any references to HSAs. Clearly, the entire Care Group structure was operated by the NGO, with little or no integration into the local healthcare structures. Even though Malawi’s integrated Care Group policy ^17^ suggests that the volunteers should receive monthly behaviour change lessons from local frontline service providers, this was clearly not happening in Dedza. The NGO coordinator explained why:

“Because we [NGO staff] support the volunteers to work with the communities on the ground, with the Care Group structure, the HSAs are taking it as the NGO’s program and not *their program*. So that mind-set also affects the sustainability… What I mean is, for example, if there is no incentive for the HSAs like the ones that are available for the volunteers, they would not be willing to work. So there should always be something like a soft drink, then they will go to work with us. They should understand that it is *their model* and we, the NGO, are just supporting them.” [61DISTRICT].

At a higher level, it was clear that District Health Officials knew the Care Groups are supposed to be integrated in the district health and nutrition activities. Supervising the Care Group activities should be an integral, and motivational, part of the District Authorities’ responsibilities. However, the resources to support these activities are often lacking, which means that district officials have to rely on the NGOs to facilitate the monitoring of the NGO-supported Care Groups:

“A: There is monitoring of Care Groups to see which ones are active; and recently the Principal Nutrition and HIV Officer was saying that any time from now we need to do that here, especially in the areas where there are no nutrition programs.
Q: How often does that happen?
A: I can’t say. Since this is my 8 ^th^ month in this job and since I came here it have never happened.
Q: But how equipped are you in terms of budget?
A: One of the problems that is making us failing to work as government nutrition officer, is lack of funds. As you might know, at District Council level, nutrition is not funded.” [59DISTRICT].

The interview data contained just one reference from a mother, suggesting that Care Groups worked with HSAs in her area [17MCB], which demonstrates that on an ad hoc basis, care group volunteers and HSAs do work together at community level. Most of the interviewees, however, did not indicate seeing HSAs and Care Group members together or appeared to associate HSAs and Care Groups. Even among the women with preterm or low-birthweight babies, there was no evidence of a coordinated follow up by Care Groups, which could have been directed by a concerned HSA. Only two out of the nine women with preterm or low-birthweight babies had received EBF support from Care Groups, and in one case this was because the local Care Group volunteer was also an aunt of the new mother who was interviewed. None of the Care Group team (paid or unpaid staff) made any reference to coordination or collaboration with HSAs or other Ministry of Health staff at District level. One of the HSAs, who described himself as a HSA supervisor, gave an elaborate description of newborn mother support provided by a mixture of HSAs and Care Group members:

“When a pregnant woman delivers, we visit the woman to see if she had any complications… When she delivers we visit her to monitor the health of the baby… if the baby or the mother has a problem, we also refer them to the hospital. From there, we explain to her how she can exclusively breastfeed her baby. So if we leave the mother, we also refer her to Care Groups… So they go and find those people in their homes and teach them how they can take care of their babies.” [40HSAB].

Yet during the interviews with the mothers, none of them appear to have received the integrated care or the referrals from HSAs to the CGVs as is described by this HSA.

Our research showed that the HSAs were not always fully informed of what was happening in the community. Whilst the HSA cadre was originally designed to be men and women who live in the rural communities they serve; evidence suggests that this is not always the case. HSAs may in fact live in a nearby town and focus primarily on tasks based at the nearby health facility or hospital whilst spending only limited amounts of time at community level. It is therefore not surprising, according to HSAs, that events such as twin or triplet births, or preterm babies brought home to a village after delivery, do not automatically come to a HSA’s attention:

“What happens at this facility is that mostly preterm babies are referred to X Hospital; that is where they are managed. And when they are being discharged they are told to pass through this health centre so that they can be linked up with a HSA from their community for follow up. So if they follow this process, they are followed up. However, there are others when they are told to pass through this health centre, they don’t, so it is difficult to follow them up.” [41HSAC].

From the perspective of the local hospitals where mothers deliver their babies and where vulnerable newborns such as premature babies or twins may spend several weeks before they are discharged, the referral system appears to be equally unclear, according to a District level interviewee:

“Q: When you have discharged a mother who has given birth to a premature baby, how do you ensure that she is receiving breastfeeding support at home?
A: We have HSAs in the community who help us with that. We do refer the babies to the HSAs. If we have their numbers, we call them but if we don’t have their contact numbers, that is now where the challenge comes in, because we don’t know what they the mothers with premature babies are really doing at home, whether they are really breastfeeding the baby or not.
Q: So you don’t have any other way of linking up with the HSAs apart from the phones?
A: No.” [60DISTRICT].

Overall, the level of support for EBF provided by any member of a professional or volunteer health workers cadre, was found to be limited, especially considering that a one in four of our interviewees were mothers of vulnerable babies, who included those in need of support with expressing breastmilk and managing this hygienically, whilst their babies needed the protection and nourishment from EBF all the more.

## Discussion

The analysis of the interview data brought to light issues regarding the efficacy of the Care Group interventions in Malawi. Our research detected no difference between the two settings with different Care Group interventions and will therefore not elaborate on any variance between them. The research highlighted that women received minimal levels of support to exclusively breastfeed their newborn babies for the first six months of their lives, and mothers with vulnerable babies were not supported in this regard. Even though Malawi is known to have high average rates of EBF among children under six months of age ( NSO & ICF, 2017), the reduction of EBF when babies are around four to five months of age, usually combined with the introduction of weaning foods ( Salim & Stones, 2020), is worrisome, as infants that young are still extremely vulnerable, and illnesses associated with contaminated water or weaning foods can have significant adverse effects.

Summary numerical responses are included to illustrate patterns and variations related to the objectives about where (exclusive) breastfeeding support was obtained, and the role of HSAs in the support for mothers with newborn babies, and in terms of HSA interaction with care group structures. In addition, the analysis of the data gave an impression of how Care Group interventions ‘sit’ within the wider system of local-level healthcare support structures in Dedza District. It demonstrated that, despite a harmonised Care Group design being put in place by Malawi’s Department of Nutrition, HIV, and AIDS in 2012 (which should allow for an integration of the NGO-supported Care Groups into the community-level government health system), such integration does not seem to be realised.

### Care Group efficacy

Our research focused on whether or not women had received breastfeeding support from members of the Care Groups working in their community, given that the Care Group members were mandated to engage with all pregnant women and women with children under two, and focus on spreading key nutrition messages, including the promotion of exclusive breastfeeding. Finding that only eight out of 26 of the women participants had received EBF support from Care Group members meant that their performance was well below expectations. Care Group interventions have often been successful in bringing women together and promoting important behaviour change messages such as those regarding breastfeeding and handwashing, which are no- or low-cost interventions that can be adopted by the large majority of women who live within the reach of Care Group activities ( Davis *et al.,* 2013; George *et al.,* 2015). The same level of success does not seem to have been achieved and sustained by the interventions focused on during this research. 

Where Dedza’s Care Groups were active, they were reported to have reinforced all-important EBF messages, but overall, it appears that breastfeeding support was not a priority for the Care Group interventions we studied. 18 out of the 26 women in areas with Care Groups reported receiving support *of* some kind (other than EBF) from care group members. In most cases this related to cooking demonstrations. Such cooking demonstrations were done in groups, as opposed to ‘passing on/receiving Care Group messages’, which the NGOs’ second tier CGVs (called ‘cluster leaders’) were expected to do on a house-to-house basis. Several references to what the community’s expectations Care Group support entails, might explain why some volunteers were reluctant to engage in visiting households empty-handed with ‘just advice’. 

While volunteer attrition is an issue that is highlighted by most researchers and practitioners of Care Groups, it was noted that the NGO that implemented the Dedza-based interventions reported high numbers of drop-out rates, in the earlier (pre-2019) phases of the Care Group interventions we studied; between 32% and 16% of the trained CGVs and/or cluster leaders reportedly becoming inactive before the end of the programme ( United Purpose, 2022). Given that this reported high attrition rate predated the COVID-19 pandemic (during which a lot of Care Group activities, including motivational visits and training sessions, had to be cancelled), we suspect that the lack of integration into the community-based HSA system may have undermined the Care Groups’ perceived legitimacy and standing within the community, thereby demotivating the volunteers. A lack of budget for supportive supervision by the district health authorities also deprived Care Group volunteers of the validation such exposure to higher level officials would bring. Research into HSA motivation shows that attrition is also a challenge among HSAs. Finding high drop-out rates is therefore not surprising ( Guenther *et al.,* 2019), yet, the budget shortage at district level that leads to a lack of opportunity to supervise and motivate both cadres is regrettable, as HSAs and CGVs could play a significant role in visiting newborns and supporting EBF, potentially saving lives. 

Another factor that may have hampered Care Group efficacy is the focus on household visits over group activities. The NGO whose project we visited explained that they interpreted Malawi’s national Care Group approach as one that requires cluster leaders to visit households on a one-to-one basis, creating significantly more work per volunteer than the traditional Care Group approach. The Malawi Care Group guidance emphasises group learning when a frontline health worker or NGO promoter passes on health, hygiene, or nutrition lessons to CGVs, but it explicitly mentions that cluster leaders should be passing on hygiene or nutrition lessons by visiting households individually. The traditional Care Group model emphasises sharing all lessons with the mothers at community level as group activities, as group meetings provide the additional benefit of creating social cohesion, the opportunity to learn from other group members and, in many cases, a sense of camaraderie Guenther *et al.,* 2019; Kok & Muula, 2013; Perera, 2015). Our realist synthesis of the Care Group literature ( Pieterse *et al.,* 2021) ^27^ uncovered that much of the CGVs’ motivation was derived from meeting with neighbourhood group members (local mothers), whose social bonds with their CGVs often provided them with affirmation and a sense of self-worth. Care Group leader visibility at community level accords status to volunteers and earns respect from peers ( Greco *et al.,* 2017). This study showed that group activities, especially the cooking demonstrations, were referred to much more often than the one-to-one visits conducted by cluster leaders, and should perhaps be prioritised, not only for cooking demonstrations but also for general knowledge sharing, and especially for passing on messages about EBF.

### A lack of collaboration between HSAs and Care Groups

The evident absence of communication and collaboration between HSAs and Care Group members can only be perceived as a missed opportunity. Given that one HSA explained in detail how a system of integrated and delegated care for women with newborn babies should have worked, there appears to be an intention on part of the HSA structures to collaborate more closely with Care Groups, but that these plans were simply not followed through on. The NGO’s programme documents also suggest that greater engagement with government structures was wished for, at District level specifically, but that government officials’ heavy workloads made scheduling meetings with the relevant individuals challenging. Similar challenges have been noted in other Care Group interventions in Malawi ( World Relief, 2004). It appears to be difficult to receive district-level buy-in for a well-integrated Care Group intervention, which translates into a lack of higher-level facilitation of the HSA-care group relationship that could have created the types of linkages that Malawi’s national Care Group strategy called for.

HSAs have been lauded with significantly expanding Malawi’s vaccine coverage, women’s access to antenatal and post-natal care and growth monitoring ( Kok & Muula, 2013; Mziray *et al.,* 2017). However, the expanding list of demands placed on HSAs have exacerbated the pressure on their time, which appears to have increased the risk of HSAs not being able to carry out all the tasks that are expected of them ( Guenther *et al.,* 2019; Kok & Muula, 2013; Ngwira *et al.,* 2021). This context, whereby HSAs are overburdened and a substantial volunteer cadre (which the Care Group volunteers effectively are) has been in place since 2012, could have lent itself to task sharing and, for example, the delegation of ‘mother and newborn baby home visits’ to care group volunteers in Malawi. There has been no evidence of this in the literature to date. Instead, the NGO staff implementing the Care Group intervention clearly struggled to convince HSAs (and those in charge of managing the HSA programme) that proper integration of the CGVs would be to the benefit of the HSAs, in terms of workload reduction, and to the benefit of women and their infants, who do currently not receive sufficient EBF and infant feeding support. Such a sharing of community-level tasks is taking place in some other LMICs, for instance in Ethiopia, where volunteer ‘women/ health development army’ members carry out a significant amount of the community-based household visits, increasing the efficiency of Ethiopia’s Health Extension Workers “in reaching households with actionable health messages” ( Yitbarek *et al.,* 2019, p 2). In a series of papers examining the latest trends in the increasing deployment of community health workers (CHWs) in LMICs, Perry *et al.* (2021) highlight an “emerging dual cadre of CHWs in which professionalized CHWs are supervising lower tiers of CHWs, who are often volunteers responsible for a small number of households”.

The lack of integration of the Care Group members into the community-based healthcare system in Malawi means that overburdened HSAs have not been given the opportunity to systematically delegate some of their home visiting responsibilities to the Care Groups, which results in a continued lack of check-ups and support for newborn babies and their mothers at community-level. In Burundi, where a randomised-controlled trial showed that Care Group-inspired interventions can be successfully adapted to be managed by Ministry of Health staff, the key to its success appears to have been the active involvement of the HSAs (or their equivalents) at community-level from the outset. This allowed the HAS to clearly see that there were potential advantages for them, in terms of being able to delegate certain time-consuming responsibilities ( Weiss *et al.,* 2015).

Our targeted search for, and reading of, all of the Government of Malawi’s health and nutrition-focused policy and strategy documents and key aid donor reports, led us to the identification of just three documents that reference Malawi’s national Care Group strategy ( GoM, DNHA, 2012; GoM, DNHA, 2017; World Bank, 2019). These include the strategy document that first included the Care Group approach, the national guidelines (in English and Chichewa, counted as one document) and a World Bank report. At the tenth anniversary of its adoption into Malawi’s national policy, it is timely to reflect on this nutrition policy component and suggest ways that can improve the efficacy of Malawi’s adapted Care Group approach. Updating and improving Malawi’s national Care Group strategy and guidance could increase the chance of community-level integration that could see Care Group members develop into a more established volunteer cadre. This would be an opportunity to conceive of CGVs as lower tier Community Health Worker (CHW) cadre, as described by Perry *et al.* (2021), who are responsible for a small number of households (similar to current CGVs) supervised by professionalised CHWs (very much like HSAs). According to the World Health Organisation, worldwide health workforce shortages are leading many low-income countries to consider ways in which CHWs can be adopted into national human resources for health strategies, inspired by examples from countries where this has been successful, for example the two-tier CHW model from Ethiopia ( Rieger *et al.,* 2019; WHO, 2020).

### Recommendations for further research

Despite there being a significant demand for care provision that is local and easily accessible, community-level healthcare provision remains a challenge in LMICs, not least because healthcare budgets are often tight. Whist there is a wide range of literature on CHW programmes, many of those are based on pilot projects, short-term projects or interventions that are donor-driven and have not been tested in terms of long-term sustainability. The Malawi government’s efforts to integrate the Care Group approach into national policy is one of the few examples where a clear intention exists to integrate lay or volunteer health worker systems into the existing community-level healthcare structures, yet until now, this approach had not been reviewed or published about. More research is needed into ways in which national-level or sub-national level efforts are made to integrate volunteer, lay and ‘stipended’ community-level healthcare providers in LMICs (such as Zulu *et al.,* 2015), as are more explorations of what shape such integrations could take in future (such as Naimoli *et al.,* 2015; Schneider & Lehmann, 2016).

### Limitations

Our study of two Care Group interventions was focused on one district in Malawi only and was based on one implementing NGO that used the same type of Care Group approach for its interventions. While this limits transferability to other Care Group interventions in Malawi, the authors of this study did further consider at least five published programme reviews and two peer reviewed articles on Care Groups in Malawi programme reviews ( Best *et al.,* 2017; Crespo *et al.,* 2009; Robins & Best, 2009; van Haeften *et al.,* 2013; World Relief, 2004), plus peer reviewed sources ( George *et al.,* 2015; Wilner *et al.,* 2017) and the findings from our realist synthesis of Care Group literature that encompasses 42 texts from a range of LMICs ( Pieterse *et al.,* 2021) in our interpretation of these findings. The focus of our study was breastfeeding, which meant that we examined the efficacy of the interventions through that specific lens, although we did consider all other mentions of our interviewees’ interactions with Care Groups. Finally, we acknowledge that we have examined interventions that are based on group interactions and people learning from face-to-face meetings to pass on behaviour change messages, in the middle of a pandemic that necessitated the cancellation of many care group activities. It can therefore be assumed that overall efficacy of the examined interventions would have been better than what we recorded, had this not been the case. However, we managed to draw most of our conclusions from a mix of field research, as well as existing programme design, policy guidelines, and pre-COVID-19 Malawi-focused research reports, on which the pandemic had no bearing.

## Conclusions

The Care Group model has been implemented in Malawi for the past 20 years and has been officially adopted as part of Malawi’s nutrition strategy since 2012. Yet, drawing on our findings in one district and further published evidence, NGOs that utilise the care group model for community-based peer learning and behaviour change interventions continue to struggle with a lack of engagement at district level. This appears to result in care groups being less integrated in the established healthcare structures than they should be, as per the existing strategy. Our research showed that the women who were interviewed received little or no dedicated support to exclusively breastfeed their babies for their full first six months. Women with vulnerable babies, born preterm or with a low birth weight, were not routinely followed up after facility discharge and only visited if a community-level HSA found out about their need for support. Where Care Groups did actively engage with women to support them with EBF and infant feeding advice, this appears to have been useful and much appreciated.

This research has highlighted several areas in which Malawi’s policy in relation to Care Groups could benefit from rationalisation. Given that community-based health workers strategies are gaining prominence in many LMICs countries, policy adaptations should be considered within this context. CGVs could be perceived as a potential cadre of individuals who can support a limited number of households each, whist integrated within, and supervised by, a professional community-level cadre, Malawi’s HSAs.

Our research, conducted a decade after the adoption of the Care Group approach into national policy in Malawi, calls for an update of the national care group guidelines, which NGOs continue to use for their Care Group interventions, and which should be able to guide district health authorities to establish their own Care Groups too. New national Care Group guidelines could take advantage of the learning that has occurred in relation to Care Group interventions in the past two decades and take on board new insights into how the adoption of a two-tier Community Health Worker approach might be able to improve access to health-related behaviour chance messages to all those who need it, and the most vulnerable especially.

## Data Availability

The transcripts of interviews for this study are restricted to protect personal data under the approval of the ethics committee of Dublin City University and the College of Medicine Research Ethics Committee (COMREC) of Malawi. Once the study was completed in March 2023, all the underlying data will be securely stored and destroyed no later than five years after the end of the project. To request access to the underlying data, researchers are required to contact the corresponding author, Pieternella Pieterse, pieternella.pieterse@dcu.ie and provide a detailed explanation as to why they wish for access to the underlying data. After the five-year period, all the data will be destroyed as noted above. OSF: Care Group research. https://doi.org/10.17605/OSF.IO/D4W2Q. ( Pieterse *et al.,* 2023). The project contains the following underlying data: Interview guidelines in English and Chichewa Codebook Care Group field research Dedza Repository: SRQR checklist for ‘Evaluating the role that Care Groups play in providing breastfeeding and infant feeding support at community level: a qualitative study in Dedza district in Malawi’. https://doi.org/10.17605/OSF.IO/D4W2Q. Data are available under the terms of the Creative Commons Zero "No rights reserved" data waiver (CC0 1.0 Public domain dedication).
