# Supplementary material for: Evaluating the role that Care Groups play in providing breastfeeding and infant feeding support at community level: a qualitative study in Dedza district in Malawi
Source: HRB Open Res. 2024 Jun 12;6:44. Originally published 2023 Sep 4. [Version 2] doi: 10.12688/hrbopenres.13736.2 (PMC11862364; doi:10.12688/hrbopenres.13736.2)
Supplement: Peer review history

# Reviewer report: Reviewer response for version 2

**DOI:** 10.21956/hrbopenres.15229.r45560

**Published:** 2025-02-25

**Authors:** Henriette Knold Rossau

**Affiliations:** 1 University of Copenhagen, Copenhagen, Denmark

Thank you for the opportunity to review this research article, which reports findings from a qualitative study exploring the role of volunteer Care Groups in Malawi in delivering breastfeeding and infant feeding support at the community level. The authors conducted 60 qualitative interviews with various stakeholders and mothers. They found that, although the intention of the volunteer Care Groups is to provide mothers with cost-effective peer-to-peer support for breastfeeding and infant feeding, very few mothers reported receiving such support. Moreover, mothers generally indicated that this type of support was less acceptable from peers compared to Health Surveillance Assistants. Another finding was that coordination between the support provided by Health Surveillance Assistants and volunteer Care Groups was almost non-existent.

**General remarks:**

 The research study is important on multiple levels. Supporting breastfeeding is crucial, especially in countries like Malawi, where weaning foods may be contaminated and pose a risk to infants. Employing volunteer support is also significant, particularly in less affluent countries. The findings from this study have the potential to inform future enhancements in breastfeeding support and care. It is essential to highlight the need for strengthening breastfeeding support and focusing on the integration and coordination of healthcare provided by lay/volunteer workers and healthcare professionals.

 The manuscript may benefit from restructuring, especially in the introduction section, and a general shortening by combining paragraphs into more concise ones. I urge the authors to cut unnecessary text from the somewhat lengthy manuscript. Additionally, ensure overall consistency in spelling (e.g., programme vs. program, Care Groups vs. care groups).

**Abstract:**

 In the background section, I propose the following alterations:

 Promoting exclusive breastfeeding is a key policy objective in Malawi. The ‘Care Group’ model, a peer-to-peer learning approach, was adopted in 2012 as a community-level policy instrument to create an integrated, multi-sectoral approach to support communities and families. Its role within community-level infant feeding support remains under-researched. This study explores the role that Care Group Volunteers (CGVs) play in providing infant feeding support at the community level.

 Due to inconsistencies in the use of assess/evaluate/explore, I propose sticking to "explore," as I believe the present study is neither an assessment nor an evaluation, but an initial exploration. However, I recommend choosing the term that best fits your study and using it consistently.

 In the findings section, I recommend revising the last sentence to reflect the qualitative nature of your empirical data. Additionally, I would refrain from stating "no evidence," as this is a stretch. Your empirical data suggested that there was little to no coordination between Health Surveillance Assistants (HSAs) and CGVs.

 I believe the conclusion can be made clearer by summarizing your findings regarding your two research objectives.

**Introduction:**

 The introduction contains a lot of detailed information that goes in different directions. See if you can consolidate the information to create a better overview. Much of the information could benefit from being made more concise. It is clear that you want to include everything, but I recommend keeping only the essential details to provide the reader with a better flow. For instance, shorten the listing of infant and neonatal mortality rates so that you don’t have to state ‘per 1,000 live births’ each time.

 I suggest mentioning breastfeeding earlier, preferably at the very beginning, to make the reader aware that this is a key topic of the paper.

 Since you state in the ‘Recommendations for further research’ section that the integration of the Care Group approach has not been evaluated and written up in detail, it might make sense to collate the information about the Care Group approach, including the mandate and implementation, in a table instead of including a lot of detail in the introduction. For me, all this information made the introduction confusing, and I would prefer a table format to ensure an easy overview of the approach, also for future referencing.

 The last paragraph of the introduction before the Aims and Objectives section needs amendment. Currently, it reads as if your paper evaluates the integration and efficacy of Care Groups, which is not the case in my perspective. First, it is sufficient to say that this has not been investigated (or published?) instead of stating that it has not been researched and published. Second, reconsider your statement about this paper addressing this knowledge gap, since your study is not an efficacy trial and does not inform about the efficacy of the Care Groups. An easy solution might be to include the word ‘part’ in the sentence.

 The paragraph ‘Study location’ should, in my opinion, be moved to the Methods section and renamed ‘Setting’.

 Unless it is required by the authors’ guidelines to address ethics (Ethical Statement) at the end of the introduction section, I would move this to the very end of the Methods section.

**Methods:**

 There is some overlap between the first two sections, ‘Study design’ and ‘Sampling and data collection’. I suggest consolidating the description of study participants under a single heading, such as ‘Study participants and sampling’. This will allow for a separate ‘Data collection’ heading to describe the procedures, starting with the sentence ‘The interviewees at district-level …’.

 The last paragraph under the data collection heading could be shortened. For example, there is no need to mention that you deleted the audio recordings after translation.

**Findings:**

 I note the quantitative approach to the reporting of qualitative data, and it is difficult to see the themes listed in Table 2 reflected in the findings. While the data analysis is stated to have followed inductive reasoning, and this is reflected in Table 2, the reporting of findings seems to follow a more deductive reasoning approach. I would like to see the findings reflect the themes in a revised version.

 There is a typo in the first sentence following the ‘Care Group interaction with HSAs and district officials’ heading: … was the examine how à was to examine how.

 Under the same heading, the fifth paragraph on p. 9 includes the same phrasing about ‘no evidence’ as I have noted in the abstract. Again, I suggest to clarify that your empirical data indicate no coordination between HSAs and CGVs.

**Discussion:**

 Is lengthy but sums up the findings nicely and puts them into context. I suggest to take a little time to see if you can make this section more concise.

 In two instances (once in the discussion and once in the conclusion) the word whilst is misspelled whist.

 Conclusion:

 Typo in the second line – terms -> term

**Figures**

 Generally, the figures are hard to read due to the low resolution. I might be the version I have, however, if it is not, I suggest to remake them using Powerpoint or another program that can accommodate nice figures.

**Figure 2:** Some of the content is illegible due to the resolution. Furthermore, the figure legend should list abbreviations so that the reader can grasp the different governmental/healthcare bodies.

Is the work clearly and accurately presented and does it cite the current literature?

Partly

If applicable, is the statistical analysis and its interpretation appropriate?

Not applicable

Are all the source data underlying the results available to ensure full reproducibility?

No source data required

Is the study design appropriate and is the work technically sound?

Yes

Are the conclusions drawn adequately supported by the results?

Yes

Are sufficient details of methods and analysis provided to allow replication by others?

Yes

Reviewer Expertise:

Complex interventions research and breastfeeding support interventions.

I confirm that I have read this submission and believe that I have an appropriate level of expertise to confirm that it is of an acceptable scientific standard, however I have significant reservations, as outlined above.

# Reviewer report: Reviewer response for version 2

**DOI:** 10.21956/hrbopenres.15229.r40618

**Published:** 2024-07-18

**Authors:** Patricia Leahy-Warren, Helen Mulcahy

**Affiliations:** 1 School of Nursing & Midwifery, University College Cork, Cork, County Cork, Ireland; 2 School of Nursing and Midwifery, National University of Ireland, Cork, Ireland

The whole document would benefit from proof reading

**Abstract**

 We note the removal of intervention related terminology in the methods section, however the results sections does not report the themes as recommended.

**Introduction**

 There is greater clarity in the introduction in terms of providing context to the study. Similarly, there is a coherence between Figure 1 and 2, in describing the input of the national care groups and where they fit within national health policy. There is a much better integration between the content in the introduction culminating in the aim of the study.

 Aims and objectives are clearly articulated and description of the study location including relevant statistics is very useful. As no data were presented on rates of breastfeeding, please comment on whether they do not exist or provide for this area.

**Methods**

  We recommended that It would be good to have an explanation as to why the village headmen were included but no changes in this regard were noted and does require a comment.

**Sampling and data collection**

 As per our previous feedback, purposive and randomization were mentioned in the abstract and the latter is more commonly used in quantitative studies. Thus, the rationale and a supporting defense of their use for qualitative research is required. Furthermore, convenience sampling is referred to under sampling and requires explanation and amendment. Table would benefit from interpretive comment after the Table so the reader is guided to the key points.

**Data Analysis**

 Reference to Taguette software needs greater clarity as either was ort was not used as opposed to ‘could’ be used. The ‘themes’ as described in Table 2, column 1 are not sufficiently labelled to be described correctly as a theme. This challenge would explain the theme names were not inserted into the abstract as recommended. For example, the first one contain ‘experience’, ‘challenges’ and ‘problems’. The references are used incorrectly in the title of the Table.

**Findings**

 The opening sentence is not appropriate under the heading findings., please revise. The sentence starting ‘key themes, should list the themes as recommended above instead the first theme appears to be drawn from Row 2 of column one of Table 2. Revisit and revise for all other themes. While the authors have made attempts to present more thematically rather than by objectives, further substantial revision is necessary if the study is to align with typical thematic analysis.

**Discussion**

 The previous recommendation regarding the structure of discussion has not yet been realized. An opening statement on the sample and who they represent is necessary. This should be followed by signposting on the direction of the discussion, e.g. it could be organized around the themes as previously recommended.

Is the work clearly and accurately presented and does it cite the current literature?

Partly

If applicable, is the statistical analysis and its interpretation appropriate?

Not applicable

Are all the source data underlying the results available to ensure full reproducibility?

Partly

Is the study design appropriate and is the work technically sound?

Partly

Are the conclusions drawn adequately supported by the results?

Partly

Are sufficient details of methods and analysis provided to allow replication by others?

Partly

Reviewer Expertise:

Prof Leahy-Warren has a h-index of 22 (Scopus)/30, with over 100 peer review publications and in excess of €4.5 M funding as Pi, co-PI, particularly focused on breastfeeding research and maternal and infant health generally.                                                                   Dr Helen Mulcahy is senior lecturer community health and has very strong educational and clinical backgrounds specific to qualitative research, parental concern, breastfeeding, primary care, community and public health nursing.

We confirm that we have read this submission and believe that we have an appropriate level of expertise to confirm that it is of an acceptable scientific standard, however we have significant reservations, as outlined above.

# Reviewer report: Reviewer response for version 2

**DOI:** 10.21956/hrbopenres.15229.r40619

**Published:** 2024-06-29

**Authors:** May Loong Tan

**Affiliations:** 1 Perinatal Research Center, School of Medicine, National Maternity Hospital, University College Dublin, Dublin, Leinster, Ireland; 2 Paediatrics, RCSI & UCD Malaysia Campus, George Town, Penang, Malaysia

Thank you for the opportunity to review the revised article. I would like to also thank the authors for taking into consideration my earlier suggestions, especially explaining the care group models. The new figures added to the necessary understanding of the background and helped make the results and discussion sections clearer.

 Other minor comments:

 Table 2: Typo "experince" - should be "experience"

 Conclusion:

 "The women included in our study received relatively little support for breastfeeding, and while the terms exclusive breastfeeding was commonly repeated by the interviewees, not all women were entirely sure what it meant." This is an interesting finding. However, I am unable to find this presented in the findings. Can you show a specific quote that demonstrated that EBF was not well understood?

Is the work clearly and accurately presented and does it cite the current literature?

Partly

If applicable, is the statistical analysis and its interpretation appropriate?

Partly

Are all the source data underlying the results available to ensure full reproducibility?

Yes

Is the study design appropriate and is the work technically sound?

Yes

Are the conclusions drawn adequately supported by the results?

Partly

Are sufficient details of methods and analysis provided to allow replication by others?

Partly

Reviewer Expertise:

Breastfeeding support, systematic review

I confirm that I have read this submission and believe that I have an appropriate level of expertise to confirm that it is of an acceptable scientific standard.

# Reviewer report: Reviewer response for version 1

**DOI:** 10.21956/hrbopenres.15025.r38173

**Published:** 2024-03-22

**Authors:** May Loong Tan

**Affiliations:** 1 Perinatal Research Center, School of Medicine, National Maternity Hospital, University College Dublin, Dublin, Leinster, Ireland; 2 Paediatrics, RCSI & UCD Malaysia Campus, George Town, Penang, Malaysia

Thank you for the opportunity to review this paper. It is a very interesting study and has a direct local impact and lessons for the broader community.  I appreciate the complexity of the study, particularly the diversity of the participants, and the area where the study was conducted. It was very easy to get confused while reading the paper because of this. Therefore, it is important that reporting is clear. Most of my comments revolve around this. There are many underlined words/phrases throughout the paper - is there a reason for them? 

 Specific comments:

 1.  Abstract:

 Only number of mothers with children under 2 years were shown, it should be consistent. Since mothers made up the majority of participants, they should be listed first. 

 "The research was conducted in one of two and several…" - this sentence could be clearer by just stating where they were conducted and not giving the numbers. It could be reworded to “The research was conducted in two care groups implementation areas and several villages where CG interventions had not taken off”. This would not only shorten the abstract but also make it clear.  The conclusion in the abstract should align with the conclusion at the end of the paper.

 2. Introduction:

 The initial part of the introduction is good as it gave a lear understanding of the setting, challenges in relation to breastfeeding support, and the history of the care groups.

 In the paragraph about the adoption of CG in Malawi, could there be more details on how the modified CG looks like. As one who is not at all familiar with this set-up, I wasn't able to visualize how the CG were implemented - which led to many questions as I continue reading.  How does the Modified Care group  differ from the original CG? A graphical representation of the CG in Malawi would be helpful. It also helps with understanding the results and the interaction between the many players within the CG.

 There seem to be 2 gaps that the researchers are trying to address, but this paper only addressed one.  The realist synthesis should be in the background instead of at the beginning of methods.

 3.  Methods

 A new word is introduced in the methods - "intervention".  What are these "interventions" and how are they different from the CG described in the background. An understanding of the CG in Dedza is needed .  The second paragraph of study design could be incorporated in the background to explain how there are different models of implementation of CG. It is not clear what are the 2 interventions. 

 Sampling  and data collection: In the abstract it was stated that the sampling of mothers was random. This needs to be described in the methods with more details of  the random sampling was done. From the current description, we know that the Village headman was first approached, who then introduced the team ti the HSA, and the HSA led the team to the mothers. How did the HSA decide which mother to pick? It sounds like a convenience sampling. 

 In the Table showing the overview of the research participants -  there should be a note to explain what is A, B and C.

 It wasn't clear if all 3 members conducted the interviews individually (if yes, what was the strategy to ensure consistency among the interviewers?). What were the roles of the 2 RA who joined the research team?

 Data analysis: "(A freely accessible software package, Taguette ( https://www.taguette.org/), could also be used to run the same analysis)"-  the purpose of this sentence is unclear. Was there a choice given to the researcher? Which was used for analysis? Was the free software package used?

 The second part of this sentence (about coding of 15% of interviews) appears to be incomplete. Suggest to reword this section.

 Table 1: Spelling error:  “audit trail” instead of “trial”. Table 1 is not very intuitive; additional text is needed to explain why is this important and relevant to the study.

 4. Results:

 In general, the results were presented like a quantitative study. There were no themes shown as one would expect from a qualitative study using thematic analysis. Sectioning the results per objective, while systematic, loses the qualitative value of the study.

 I understand the authors' intention to show the numbers, but these numbers need to be interpreted with much caution. The study wasn't designed as such therefore, the quantitative results should be limited.

 Figure 1: While it does complement the paper, it should not be the star.

 The themes were not clearly presented. The qualitative analysis was thematic analysis but results did not present the themes.

 5. Discussion:

 This section was able to draw together the findings presented in the results section. The section could start with a summary of the findings (see my comment in Conclusion). One thing missing from the discussion is the potential changes to be implemented if CGs are still relevant to the community. What further actions has been taken or should be taken?

 6. Conclusion: This section is too long. The first paragraph is a summary of the results and would fit better as the first paragraph of the discussion.

 The conclusion here must match the conclusion in the abstract.

Is the work clearly and accurately presented and does it cite the current literature?

Partly

If applicable, is the statistical analysis and its interpretation appropriate?

Partly

Are all the source data underlying the results available to ensure full reproducibility?

Yes

Is the study design appropriate and is the work technically sound?

Yes

Are the conclusions drawn adequately supported by the results?

Partly

Are sufficient details of methods and analysis provided to allow replication by others?

Partly

Reviewer Expertise:

Breastfeeding support, systematic review

I confirm that I have read this submission and believe that I have an appropriate level of expertise to confirm that it is of an acceptable scientific standard, however I have significant reservations, as outlined above.

# Response

**Published:** 2024-04-05

**Authors:** Pieternella Pieterse

**Affiliations:** Science and Health, Dublin City University, Glasnevin, Dublin, Ireland

Dear Dr May Loong Tan, on behalf of the research team, I would like to thank you for taking the time to review this article and provide extensive feedback. We have taken your feedback on board, along with that of reviewer 1, and have created an updated version of the article, which we believe is much improved. Thank you!

 Specific comments:

 1.  Abstract:

 Only number of mothers with children under 2 years were shown, it should be consistent. Since mothers made up the majority of participants, they should be listed first. 

 "The research was conducted in one of two and several…" - this sentence could be clearer by just stating where they were conducted and not giving the numbers. It could be reworded to “The research was conducted in two care groups implementation areas and several villages where CG interventions had not taken off”. This would not only shorten the abstract but also make it clear.  The conclusion in the abstract should align with the conclusion at the end of the paper.

*[ **response: **We agree and the abstract has now been updated with this feedback in mind]*

 2. Introduction:

 The initial part of the introduction is good as it gave a clear understanding of the setting, challenges in relation to breastfeeding support, and the history of the care groups.

 In the paragraph about the adoption of CG in Malawi, could there be more details on how the modified CG looks like. As one who is not at all familiar with this set-up, I wasn't able to visualize how the CG were implemented - which led to many questions as I continue reading.  How does the Modified Care group differ from the original CG? A graphical representation of the CG in Malawi would be helpful. It also helps with understanding the results and the interaction between the many players within the CG.

*[ **response: **we agree with this suggestion and have now added two illustrations to the paper that should be able to demonstrate the difference.]*

 There seem to be 2 gaps that the researchers are trying to address, but this paper only addressed one. 

*[ **response: **We have made the objectives clearer and merged the original objective 1-2. We have also ensured that we refer to both of the new objectives in the concluding section of the paper.*

 The realist synthesis should be in the background instead of at the beginning of methods.

*[ **response: **we agree and a short synopsis of the findings of the realist synthesis is now included in the background]*

 3.  Methods

 A new word is introduced in the methods - "intervention".  What are these "interventions" and how are they different from the CG described in the background. An understanding of the CG in Dedza is needed .  The second paragraph of study design could be incorporated in the background to explain how there are different models of implementation of CG. It is not clear what are the 2 interventions. 

*[ **response: **Some of this information about the Care Group interventions is more than needed to describe the study and this has now been removed, some details have been moved into the background section, as you suggest.]*

 Sampling  and data collection: In the abstract it was stated that the sampling of mothers was random. This needs to be described in the methods with more details of  the random sampling was done. From the current description, we know that the Village headman was first approached, who then introduced the team to the HSA, and the HSA led the team to the mothers. How did the HSA decide which mother to pick? It sounds like a convenience sampling. 

*[ **response:** we agree, convenience sampling was used, again this section will be clarified]*

 In the Table showing the overview of the research participants -  there should be a note to explain what is A, B and C.

*[ **response:** The table is confusing so it has been removed, the necessary info has been described instead.]*

 It wasn't clear if all 3 members conducted the interviews individually (if yes, what was the strategy to ensure consistency among the interviewers?). What were the roles of the 2 RA who joined the research team?

*[ **response: **This will be clarified in the text; the co-PI of the project and all four others (incl the two RAs) conducted the interviews. Because of a limited budget for transport and limited time in the team’s teaching schedule, this was deemed the best way to collect the data. We (all team members, including those based in Ireland) jointly compiled the interview guidelines using Zoom calls and emails, which were translated by the team members. All data collectors carried copies of the interview guides – some of this will be clarified in the text]*

 Data analysis: "(A freely accessible software package, Taguette ( https://www.taguette.org/), could also be used to run the same analysis)"-  the purpose of this sentence is unclear. Was there a choice given to the researcher? Which was used for analysis? Was the free software package used?

*[ **response:** It is HRB policy that when authors refer to certain paid-for software if referenced (in this case Nvivo, which we used) that a free alternative is also referenced, so we added this line on HRB’s request, we did not use this software.]*

 The second part of this sentence (about coding of 15% of interviews) appears to be incomplete. Suggest to reword this section.

*[ **response:** thanks, this has now been fixed]*

 Table 1: Spelling error:  “audit trail” instead of “trial”. Table 1 is not very intuitive; additional text is needed to explain why is this important and relevant to the study.

*[ **response:** We were asked to include this table to demonstrate that we had done enough to ensure the trustworthiness of this study. It has been moved to a more appropriate location and we have uploaded an image of the table rather than a word file, which did not download well when we tried]*

 4. Results:

 In general, the results were presented like a quantitative study. There were no themes shown as one would expect from a qualitative study using thematic analysis. Sectioning the results per objective, while systematic, loses the qualitative value of the study.

*[ **response:** we agree, a table of key themes has now been added to the findings, and the findings chapter has been reworked to remove some of the focus on the numbers, and to bring out the themes and responses]*

 I understand the authors' intention to show the numbers, but these numbers need to be interpreted with much caution. The study wasn't designed as such therefore, the quantitative results should be limited.

 Figure 1: While it does complement the paper, it should not be the star.

*[ **response:** the table has now been removed]*

 The themes were not clearly presented. The qualitative analysis was thematic analysis but results did not present the themes.

*[ **response:** a table with the themes has now been added at the end of the data analysis section and the themes have now been given more prominence in the text]*

 5. Discussion:

 This section was able to draw together the findings presented in the results section. The section could start with a summary of the findings (see my comment in Conclusion). One thing missing from the discussion is the potential changes to be implemented if CGs are still relevant to the community. What further actions has been taken or should be taken?

*[ **response: **Thanks, this will be added (even though it is a difficult question to answer)]*

 6. Conclusion: This section is too long. The first paragraph is a summary of the results and would fit better as the first paragraph of the discussion.

 The conclusion here must match the conclusion in the abstract.

*[ **response: **Thanks, we have taken these comments on board also, and moved the first paragraph to the discussion section. The abstract has been rewritten and does now match better with the conclusion]*

# Reviewer report: Reviewer response for version 1

**Published:** 2023-12-20

**Authors:** Patricia Leahy-Warren, Helen Mulcahy

**Affiliations:** 1 School of Nursing & Midwifery, University College Cork, Cork, County Cork, Ireland; 2 School of Nursing and Midwifery, National University of Ireland, Cork, Ireland

Thank you for the opportunity to review your manuscript as breastfeeding is a very important public health matter regardless of country and continent. Learnings from the experience of individual countries are a valuable contribution to enhancing breastfeeding policy and strategy internationally. Overall, we believe the manuscript would benefit from revisiting the structure and presentation to better align with the steps of the research process. 

**The abstract**

 The title, aim and design of study need to be made explicit and be consistent throughout. The abstract provides too much irrelevant details in the methods section and should instead state the design, the setting and the final sample, without providing confusing numbers. 

 The results need to describe the themes identified. Furthermore, given the current title relates to evaluation, it is unclear why you would select an area where the intervention had not started.

 In general, it is best to use language relevant to qualitative inquiry rather than numbers and the conclusion needs to reflect the findings.

**Introduction**

 In terms of up to date literature, the authors' previous realist synthesis was cited but it would have been useful to have a summary within this paper to have context for the reader. It would be useful to introduce how many African mothers are undermined in relation to the value of EBM by the lack of legislation in relation to the marketing of infant formula. This could be positioned after the reference Guenther et al., 2019.

 On page 3 and throughout, your underlining needs to be explained to the reader. Consider editing page 3 to be more succinct and focused on breastfeeding. The final paragraph leading into the Aim, would suggest that a systematic review is following so this requires revision to lead in to the study.

 The section entitled 'aims and objectives' needs to be revised as there needs to be coherence between the title, aim and study design. The findings of this review (Pieterse et al., 2021) needs to be integrated into the above literature/background. 

**Methods**

 A 'qualitative approach' is a research paradigm and a specific research design needs to be explained, and rationalized with supporting references.

 It would be good to have an explanation as to why the village headmen were included.

 The  two paragraphs preceding 'research setting' should be edited and integrated into the background section.

 Within the section starting with '... 20% up to ...19 to 29', should be revised and edited and placed appropriately within the background.

 The sentence beginning '...In Dedza, 18%...' needs to be made explicit in the context of sampling.

**Sampling and Data Collection**

 The first sentence from '...to explore how and from....to '...most recent baby/ies...' needs to be revised for clarity.

 This section needs to include: who are the population of interest, what is the sampling strategy and how is it defended, how many were recruited and how  many participated. Purposive and randomization were mentioned in the abstract and the latter is more commonly used in quantitative studies. Thus the rationale and a supporting defense of their use for qualitative research is required.

 Data collection needs to more succinctly described and summarized, including the interview guide rather than referring the reader to another paper. The reader needs to know what were they asked.

**Data Analysis**

 The first lengthy sentence requires clarity as does the use of brackets.

 The order in which data analysis was done is not clear. Describe data analysis as per the process and end with leading into key themes. The Table in terms of trustworthiness is inappropriately positioned within the paper and also requires text of description and interpretation. 

**Results**

 Use of the term findings are more applicable to qualitative studies and you may consider changing. This section needs to have an opening sentence to present the characteristics of the sample followed by the themes and how they addressed the objectives of the study. Rather than presenting in question and answer format, the themes need to be used to better effect to summarize and interpret the data from the themes rather than the objectives. What does the underlining of some text mean for the reader? Reference to policy within the results section causes confusion and should be integrated within the discussion.  

**Discussion**

 This section would benefit from an opening statement on the sample and who they represent. This should be followed by signposting on the direction of the discussion, e.g. it could be organized around the themes.

 Reference to numerical responses at the discussion stage needs to be presented earlier and it needs to be clarified here in the discussion. Then specify the patterns and variations that were found and how these compare to previous literature or are these novel findings. The concept of efficacy in this context needs to be explained earlier in the paper and also how this was measured. The discussion needs to be edited to focus on the themes and your findings. Then compare and contrast these with previous literature. Highlight novel results from this study in making an important contribution to the body of knowledge. 

 The paragraph immediately prior to recommendations seems inappropriately placed and like above all findings need to be discussed in the context of empirical and also relevant grey literature  (e.g. Malawi's Department of Health). Recommendations need to start with your study findings so that they are specifically emerging from your findings. 

 Prof Patricia Leahy-Warren

 Dr Helen Mulcahy 

 School of Nursing and Midwifery, University College, Cork

Is the work clearly and accurately presented and does it cite the current literature?

Partly

If applicable, is the statistical analysis and its interpretation appropriate?

Not applicable

Are all the source data underlying the results available to ensure full reproducibility?

Partly

Is the study design appropriate and is the work technically sound?

Partly

Are the conclusions drawn adequately supported by the results?

Partly

Are sufficient details of methods and analysis provided to allow replication by others?

Partly

Reviewer Expertise:

Prof Leahy-Warren has a h-index of 22 (Scopus)/30, with over 100 peer review publications and in excess of €4.5 M funding as Pi, co-PI, particularly focused on breastfeeding research and maternal and infant health generally.                                                                   Dr Helen Mulcahy is senior lecturer community health and has very strong educational and clinical backgrounds specific to qualitative research, parental concern, breastfeeding, primary care, community and public health nursing.

We confirm that we have read this submission and believe that we have an appropriate level of expertise to confirm that it is of an acceptable scientific standard, however we have significant reservations, as outlined above.

# Response

**Published:** 2024-04-05

**Authors:** Pieternella Pieterse

**Affiliations:** Science and Health, Dublin City University, Glasnevin, Dublin, Ireland

Dear Prof Leahy-Warren and Dr  Mulcahy. On behalf of the research team, we would like to thank you for taking the time to provide the extensive feedback this article needed. As you can see below, we have taken all of your comments and suggestions on board as well as that from reviewer 2. This, we believe has much improved the article. Once again thanks. 

**The abstract**

 The title, aim and design of study need to be made explicit and be consistent throughout. The abstract provides too much irrelevant details in the methods section and should instead state the design, the setting and the final sample, without providing confusing numbers. 

 The results need to describe the themes identified. Furthermore, given the current title relates to evaluation, it is unclear why you would select an area where the intervention had not started.

 In general, it is best to use language relevant to qualitative inquiry rather than numbers and the conclusion needs to reflect the findings.

*[response: we agree with many of the observations; the abstract has been rewritten with this feedback in mind]*

**Introduction**

 In terms of up to date literature, the authors' previous realist synthesis was cited but it would have been useful to have a summary within this paper to have context for the reader.

*[response: a summary has now been added]*

 It would be useful to introduce how many African mothers are undermined in relation to the value of EBM by the lack of legislation in relation to the marketing of infant formula. This could be positioned after the reference Guenther et al., 2019.

*[response: we agree and have added a reference to this, in the paragraph before the one you indicated just before the reference by Victora et al 2016]*

 On page 3 and throughout, your underlining needs to be explained to the reader.

*[response: the underlining has been removed]*

 Consider editing page 3 to be more succinct and focused on breastfeeding. The final paragraph leading into the Aim, would suggest that a systematic review is following so this requires revision to lead into the study.

*[response: The wording has now been changed]*

 The section entitled 'aims and objectives' needs to be revised as there needs to be coherence between the title, aim and study design.

*[response: the study title and the aim are now the exact same]*

 The findings of this review (Pieterse et al., 2021) needs to be integrated into the above literature/background. 

*[response: a summary of this study has now been added]*

**Methods**

 A 'qualitative approach' is a research paradigm and a specific research design needs to be explained, and rationalized with supporting references.

*[response: agreed, we have done so]*

 It would be good to have an explanation as to why the village headmen were included.

*[response: the text already included the following text: “To examine the role that healthcare providers, community leaders and district health officials’ play in supporting and integrating Care Groups into the communities, we interviewed village headmen, HSAs and a number of district- level health officials, as well as an NGO representative.” Our Malawi-based research team designed the data collection protocol, which started with a curtsey call to the village headman in each community, then an introduction to the HSAs, etc. - we included the data collected from all individuals.*

 The  two paragraphs preceding 'research setting' should be edited and integrated into the background section.

*[response: we agree and this has move been moved.]*

 Within the section starting with '... 20% up to ...19 to 29', should be revised and edited and placed appropriately within the background.

 The sentence beginning '...In Dedza, 18%...' needs to be made explicit in the context of sampling.

*[response: we agree, the entire section about the study location has now been moved up to before the methods section]*

**Sampling and Data Collection**

 The first sentence from '...to explore how and from....to '...most recent baby/ies...' needs to be revised for clarity.

 This section needs to include: who are the population of interest, what is the sampling strategy and how is it defended, how many were recruited and how  many participated. Purposive and randomization were mentioned in the abstract and the latter is more commonly used in quantitative studies. Thus the rationale and a supporting defense of their use for qualitative research is required.

*[response: we agree and this has now been addressed, and we believe that we should call the sampling method used 'convenience sampling']*

 Data collection needs to more succinctly described and summarized, including the interview guide rather than referring the reader to another paper. The reader needs to know what were they asked.

*[response: we agree, the methods section has been revised significantly to clarify the issues you have highlighted above.]*

**Data Analysis**

 The first lengthy sentence requires clarity as does the use of brackets.

*[response: this has now been adjusted. HRB’s requirement of including information on a freely available software package that could be used instead of the software mentioned, adds to the confusion.]*

 The order in which data analysis was done is not clear. 

*[response: this paragraph was tidied up]*

 Describe data analysis as per the process and end with leading into key themes.

*[response: A table with the key themes has now been added]*

 The Table in terms of trustworthiness is inappropriately positioned within the paper and also requires text of description and interpretation. 

*[response: this table has now been moved to a more appropriate place]*

**Results**

 Use of the term findings are more applicable to qualitative studies and you may consider changing.

*[response: we agree, this has now been changed to findings]*

 This section needs to have an opening sentence to present the characteristics of the sample followed by the themes and how they addressed the objectives of the study.

*[response: we agree, we have added an opening sentence and have rewritten this section]*

 Rather than presenting in question and answer format, the themes need to be used to better effect to summarize and interpret the data from the themes rather than the objectives.

*[Response: this part of the paper has been rewritten and the themes have more prominence now]*

 What does the underlining of some text mean for the reader?

*[Response: the underling has been removed]*

 Reference to policy within the results section causes confusion and should be integrated within the discussion.  

*[response: this has now been moved]*

**Discussion**

 This section would benefit from an opening statement on the sample and who they represent. This should be followed by signposting on the direction of the discussion, e.g. it could be organized around the themes.

*[response: both have been added]*

 Reference to numerical responses at the discussion stage needs to be presented earlier and it needs to be clarified here in the discussion. Then specify the patterns and variations that were found and how these compare to previous literature or are these novel findings. The concept of efficacy in this context needs to be explained earlier in the paper and also how this was measured. The discussion needs to be edited to focus on the themes and your findings. Then compare and contrast these with previous literature. Highlight novel results from this study in making an important contribution to the body of knowledge. 

*[response: the section has been rewritten with this feedback in mind]*

 The paragraph immediately prior to recommendations seems inappropriately placed and like above all findings need to be discussed in the context of empirical and also relevant grey literature  (e.g. Malawi's Department of Health). Recommendations need to start with your study findings so that they are specifically emerging from your findings. 

*[response: this paragraph has been shortened to link more clearly with the study findings; which are, in part, focused on Malawi’s integrated Care Group model not working so well as a type of lay/ health extension worker model]*
